# Is chronic malnutrition associated with an increase in malaria incidence? A cohort study in children aged under 5 years in rural Gambia

**DOI:** 10.1186/s13071-018-3026-y

**Published:** 2018-08-06

**Authors:** Anne L. Wilson, John Bradley, Ballah Kandeh, Kolawole Salami, Umberto D’Alessandro, Margaret Pinder, Steven W. Lindsay

**Affiliations:** 1 Department of Biosciences, Durham University, Stockton Road, Durham, DH1 3LE UK; 2 Medical Research Council Tropical Epidemiology Group, Department of Infectious Disease Epidemiology, Faculty of Epidemiology and Population Health, London School of Hygiene and Tropical Medicine, Keppel Street, London, WC1E 7HT UK; 3 National Malaria Control Programme, Banjul, The Gambia; 4 Medical Research Council Unit The Gambia at the London School of Hygiene and Tropical Medicine, Banjul, The Gambia; 5 Department of Disease Control, Faculty of Infectious and Tropical Diseases, London School of Hygiene and Tropical Medicine, Keppel Street, London, WC1E 7HT UK

**Keywords:** Malaria, Malnutrition, Stunting, Anaemia, Children, The Gambia, Sub-Saharan Africa

## Abstract

**Background:**

Malnutrition is common in children in sub-Saharan Africa and is thought to increase the risk of infectious diseases, including malaria. The relationship between malnutrition and malaria was examined in a cohort of 6–59 month-old children in rural Gambia, in an area of seasonal malaria transmission. The study used data from a clinical trial in which a cohort of children was established and followed for clinical malaria during the 2011 transmission season. A cross-sectional survey to determine the prevalence of malaria and anaemia, and measure the height and weight of these children was carried out at the beginning and end of the transmission season. Standard anthropometric indices (stunting, wasting and underweight) were calculated using *z*-scores.

**Results:**

At the beginning of the transmission season, 31.7% of children were stunted, 10.8% wasted and 24.8% underweight. Stunting was more common in Fula children than other ethnicities and in children from traditionally constructed houses compared to more modern houses. Stunted children and underweight children were significantly more likely to have mild or moderate anaemia. During the transmission season, 13.7% of children had at least one episode of clinical malaria. There was no association between stunting and malaria incidence (odds ratio = 0.79, 95% CI: 0.60–1.05). Malaria was not associated with differences in weight or height gain.

**Conclusions:**

Chronic malnutrition remains a problem in rural Gambia, particularly among the poor and Fula ethnic group, but it was not associated with an increased risk of malaria.

**Trial registration:**

Trial registration: ISRCTN, ISRCTN01738840, registered: 27/08/2010 (Retrospectively registered).

## Background

In sub-Saharan Africa (SSA), children under five years are the most vulnerable to malaria and malnutrition [1, 2]. Despite the positive impact of the scale-up of control interventions [3], malaria remains a major burden, with 194 million malaria cases and 407,000 malaria deaths in Africa in 2016 [1]. Malnutrition is a multi-factorial problem which is caused by the combined effects of poverty, food insecurity, lack of diversity in the diet, infectious diseases such as diarrhoea, low access to clean water and sanitation, and poor understanding of nutrition and hygiene [4, 5]. Stunting, defined as low height for age, is a measure of chronic malnutrition and results from poor nutrition in early childhood which leads to failure to grow, both physically and cognitively. Stunting can have deleterious effects on school achievement and economic productivity in adult life [6]. While the prevalence of stunting has been declining slowly in SSA, the absolute number of stunted children under five years of age has increased by 17% from 2000, with an estimated 59 million children stunted in 2016 [2]. Wasting, defined as low weight for height, occurs as a result of rapid weight loss or failure to gain weight and can lead to weakened immunity, long term developmental delays and increased risk of death. Fourteen million African children under five years of age were wasted in 2016 (7.4%), of which 4.1 million were severely wasted [2].

There is a vicious cycle between childhood nutrition and infections. Poor nutritional status can suppress immunity, resulting in an increased risk and poor prognosis of infections, and continued infections can lead to declining nutritional status [7, 8]. The relationship between malnutrition and malaria and *vice versa* is difficult to elucidate and studies have shown contradictory results. For example, some studies have demonstrated an increased risk of malaria associated with malnutrition [9, 10] whilst others report a decreased risk [11] or no effect [12–16]. Several studies reported lower anti-*Plasmodium falciparum* specific antibodies in malnourished children compared to those with normal anthropometric values [11, 17, 18], although other studies did not [19, 20]. A recent systematic review found that anthropometric parameters were not associated with malaria incidence or parasite density [21]. Studies looking at the effect of malaria on malnutrition have also not shown consistent results [9, 22–24] and a systematic review concluded there was no association [25]. Intervention studies, however, indicate that malaria control measures are associated with improved nutritional status in children [26–29].

This study aimed to determine the prevalence of malnutrition in children aged between 6 and 59 months living in rural Gambia, where malaria is highly seasonal, and its association with malaria and anaemia. There were two main objectives: one was to determine whether stunting at the beginning of the malaria transmission season was associated with an increased incidence of malaria, and the other to understand whether malaria during the transmission season had any influence on weight and height gain. Identification of an association between malaria and nutritional status or *vice versa* would strengthen the case for action against these debilitating health conditions.

## Methods

### Study site

The study was conducted in the Upper River Region (URR) (regional capital: Basse Sante Su, 13°19'0.12"N, 14°13'0.12"W), a rural area of open Sudanian savannah. The URR is divided into north and south banks by the River Gambia. Residents on the south bank are typically more affluent than on the north bank. Malaria transmission is highly seasonal and associated with the annual rains which occur from June to October. The rainy season is also called the “hungry season” as it corresponds to the end of food supplies from the previous harvest before the new harvesting period [30].

### Data collection

The study used data from a cluster-randomised trial which assessed whether long-lasting insecticidal nets (LLINs) and indoor residual spraying (IRS) provide better protection against malaria in children than LLINs alone. The study design and results are described elsewhere [31, 32]. Briefly, 70 clusters of villages were randomised to receive LLINs and IRS, or LLINs alone. Permethrin-treated LLINs (2%; Olyset Nets, Sumitomo Chemicals, Tokyo, Japan) were distributed in both arms at the start of the 2010 transmission season. IRS with DDT (2 g/m^2^, DDT 75% wettable powder; Hindustan Insecticides, New Delhi, India) was applied to dwelling rooms at the start of each transmission season in the IRS-LLIN arm.

This sub-study obtained additional written consent from the caregivers of children aged 6–59 months to collect anthropometric measures during the second year (2011) of the trial. Malaria episodes during the transmission season were identified by passive case detection. Children were surveyed at the beginning (June 2011) and end (January 2012) of the transmission season to determine the prevalence of *P. falciparum* infections using microscopy, anaemia [haemoglobin level (g/dl)] using spectrophotometers (HemoCue® Hb 301 System, HemoCue, Ängelholm, Sweden), and measure weight and height. The weight of each child was measured by two fieldworkers using hanging scales that were recalibrated frequently. Children under 2 years-old had their recumbent height measured, whilst older children were measured standing using a stadiometer (Leicester Portable Height Measure). Socio-demographic factors such as gender, ethnicity, housing construction (roof and wall material), village, bank of the river the village was located on, use of an LLIN the previous night and study arm (house treated with IRS or not) were collected at the first survey.

### Outcome definitions

Clinical malaria was defined as an axillary temperature of ≥ 37.5 °C and/or a history of fever in the past 48 h, and a positive rapid diagnostic test (Paracheck Pf Device, Orchid Biomedical Systems, Goa, India). After a malaria episode, the child was not considered at risk of malaria for the next 28 days. Anthropometric indices were expressed in relation to the World Health Organization (WHO) child growth standards [33] and were calculated using the WHO Anthro macro for Stata (version 3.2.2, January 2011). Stunting, wasting, and underweight were defined as height-for-age, weight-for-length, and weight-for-age of less than 2 standard deviations (*z*-scores) below the WHO growth standard reference mean. *Z-*scores of more than 4 or less than -4 were excluded. Mild, moderate and severe anaemia were defined as a haemoglobin level of less than 11, 8 and 5 g/dl, respectively. Houses were said to be traditional if more than 50% of the rooms had mud walls and a thatched roof, and modern if otherwise.

### Data analysis

The dataset was restricted to children present at both cross-sectional surveys. Children who were 5 years or older were excluded from the analysis of the second cross-sectional survey (since *z*-scores calculated against WHO child growth standards are only valid up to this age).

The prevalence of *P. falciparum* infection, anaemia, stunting, wasting and underweight was calculated for each cross-sectional survey. Socio-demographic risk factors for stunting, wasting and underweight were identified. Chi-square tests were used to compare proportions. Logistic regression with a random effect for village was used to determine risk factors for at least one malaria episode, including stunting at the start of the transmission season, while adjusting for confounding factors. Linear mixed models were used to determine the association between weight/height/haemoglobin concentration at the end of season survey and incidence of at least one malaria episode, adjusting for baseline weight/height/haemoglobin concentration and confounding variables, with cluster as a random effect. Statistical analysis was conducted using Stata Statistical Software (Release 14, StataCorp. 2015, College Station, TX, USA).

## Results

A total of 2527 children aged between 6 and 59 months were surveyed at the first cross-sectional survey. The mean age of enrolment was 2.79 years (interquartile range = 2.20) and children were predominantly of Fula (42.5%) or Mandinka (47.7%) ethnicity (Table 1). The trial achieved a high coverage of LLINs (93.6%).

**Table 1:** Characteristics of study children at the start of the transmission season, June 2011

Characteristic of children / household (*N* = 2527)	
Mean age (years) at enrolment (interquartile range)	2.79 (2.20)
Age at enrolment, *n* (%)	
6 months to < 1 year	273 (10.8)
1 year to < 2 years	523 (20.7)
2 years to < 3 years	593 (23.5)
3 years to < 4 years	548 (21.7)
4 years to < 5 years	590 (23.3)
Female, *n* (%)	1179 (46.7)
Ethnicity, *n* (%)	
Fula	1073 (42.5)
Mandinka	1205 (47.7)
Other	245 (9.7)
Village located on North bank, *n* (%)	1303 (51.6)
Traditional house^a^, *n* (%)	1073 (42.5)
Sleeping under any net, *n* (%)	2383 (94.3)
Sleeping under a long-lasting insecticidal net, *n* (%)	2366 (93.6)
House sprayed with DDT, *n* (%)	1269 (50.2)

^a^At least half of the rooms in household have mud walls and thatched roof

Comparing clinical characteristics of the children between the two cross-sectional surveys, children were significantly less likely to be wasted or underweight at the end of season survey (wasting = 8.3%; underweight = 20.3%) compared to the baseline survey (wasting = 10.8%, *P* = 0.004; underweight = 24.8%, *P* < 0.001) (Table 2). However, a similar proportion of children were stunted at the first (31.7%) and second survey (33.7%). Prevalence of parasitaemia was significantly higher at the second (13.5%) than the first survey (5.5%, *P* < 0.001), while the mean haemoglobin level was significantly lower at the end (mean Hb = 10.4 g/dl, 95% CI: 10.3–10.4) than at the beginning of the transmission season (mean Hb = 10.5 g/dl, 95% CI: 10.5–10.6, *P* < 0.001). Prevalence of mild anaemia was the same between surveys (both 58.4%), though moderate anaemia was significantly higher at the end of the transmission season (8.9% of children *vs* 6.0% at baseline, *P* < 0.001). No severe anaemia cases were found at the beginning of the transmission season, while 6 children (0.2%) were severely anaemic at the end.

**Table 2:** Anthropometric and malariometric characteristics of study children at the start and end of transmission season surveys

Characteristics of study children (*N* = 2527)	Start of transmission season survey	End of transmission season survey	*P*-value
Mean weight (95% CI) (kg)	11.6 (11.5–11.7)	13.1 (13.0–13.3)	<0.001
Mean height (95% CI) (cm)	87.5 (87.0–87.9)	91.9 (91.5–92.4)	<0.001
Stunted^a^, *n* (%)	796/2511 (31.7)	725/2152 (33.7)	0.1
Wasted^a^, *n* (%)	272/2508 (10.8)	177/2126 (8.3)	0.004
Underweight^a^, *n* (%)	624/2516 (24.8)	437/2158 (20.3)	<0.001
*P. falciparum* parasitaemia	139 (5.5)	341 (13.5)	<0.001
Mean haemoglobin (95% CI) (g/dl)	10.5 (10.5–10.6)	10.4 (10.3–10.4)	<0.001
Mild anaemia (Hb < 11 g/dl), *n* (%)	1476 (58.4)	1476 (58.4)	1
Moderate anaemia (Hb < 8 g/dl), *n* (%)	151 (6.0)	225 (8.9)	<0.001
Severe anaemia (Hb < 5 g/dl), *n* (%)	0	6 (0.2)	–

^a^Children with *z*-scores of more than 4 or less than -4 are not included and anthropometric data from children aged > 5 years at end of transmission survey were censored

During the first survey, children that were wasted or underweight were significantly younger. The mean age was 30.0 months (95% CI: 28.2–31.9) for wasted children and 34.4 months (95% CI: 33.8–35.1) for non-wasted ones (*P* < 0.001). Similarly, the mean age was 32.7 months (95% CI: 31.6–33.9) for underweight children and 34.4 months (95% CI: 33.7–35.1) for non-underweight ones (*P* = 0.02). There was no significant difference in the mean age of stunted (33.2 months, 95% CI: 32.2–34.1) and non-stunted children (34.3 months, 95% CI: 33.5–35.1, *P* = 0.09). Ethnicity was strongly associated with malnutrition, with Fula children more likely to be stunted (40% stunting prevalence in Fula *vs* 24.7% in Mandinka, *P* < 0.001) and underweight (30.1% underweight prevalence in Fula *vs* 19.6% in Mandinka, *P* < 0.001) than Mandinka children (Table 3). There was no significant difference in the prevalence of wasting by ethnicity. Living in a traditional house was also associated with stunting, with 37.1% of children living in traditional houses stunted compared to 27.7% of children living in more modern houses (*P* < 0.001). Living in a traditional house was not significantly associated with wasting or underweight.

**Table 3:** Bi-variable analysis of factors associated with malnutrition at the start of the malaria transmission season

Risk factor	Stunting (*N* = 2511)			Wasting (*N* = 2508)			Underweight (*N* = 2516)		
	Prevalence *n*/*N* (%)	Odds ratio^b^ (95% CI)	*P*-value	Prevalence *n*/*N* (%)	Odds ratio^b^ (95% CI)	*P*-value	Prevalence *n*/*N* (%)	Odds ratio^b^ (95% CI)	*P*-value
Gender									
Male	420/1338 (31.4)	1		157/1337 (11.7)	1		331/1341 (24.7)	1	
Female	376/1173 (32.1)	1.06 (0.89–1.26)	0.5	115/1171 (9.8)	0.81 (0.63–1.05)	0.1	293/1175 (24.9)	1.03 (0.86–1.24)	0.8
Ethnicity									
Fula	426/1066 (40.0)	1		125/1068 (11.7)	1		322/1071 (30.1)	1	
Mandinka	297/1201 (24.7)	0.49 (0.40–0.61)	<0.001	117/1194 (9.8)	0.81 (0.60–1.09)	0.2	235/1198 (19.6)	0.56 (0.45–0.70)	<0.001
Others	71/240 (29.6)	0.63 (0.45–0.90)	0.01	29/242 (12.0)	1.03 (0.64–1.65)	0.9	66/243 (27.2)	0.86 (0.60–1.22)	0.4
Village location									
South bank	396/1220 (32.5)	1		136/1218 (11.2)	1		310/1222 (25.4)	1	
North bank	400/1291 (31.0)	0.93 (0.71–1.22)	0.6	136/1290 (10.5)	0.94 (0.71–1.25)	0.7	314/1294 (24.3)	0.95 (0.74–1.22)	0.7
House construction									
Modern	400/1445 (27.7)	1		169/1442 (11.7)	1		347/1447 (24.0)	1	
Traditional^a^	396/1066 (37.1)	1.44 (1.19–1.73)	<0.001	103/1066 (9.7)	0.81 (0.62–1.06)	0.1	277/1069 (25.9)	1.04 (0.86–1.27)	0.7
Slept under an LLIN									
No	3/16 (18.8)	1		3/16 (18.8)	1		5/16 (31.3)	1	
Yes	749/2351 (31.9)	1.91 (0.53–6.95)	0.3	249/2349 (10.6)	0.49 (0.14–1.77)	0.3	579/2356 (24.6)	0.73 (0.24–2.16)	0.6
House received DDT-IRS									
No	396/1248 (31.7)	1		126/1251 (10.1)	1		306/1254 (24.4)	1	
Yes	400/1263 (31.7)	0.97 (0.74–1.27)	0.8	146/1257 (11.6)	1.16 (0.87–1.54)	0.3	318/1262 (25.2)	1.00 (0.78–1.29)	1.0

^a^At least 50% of rooms with mud walls and thatched roof

^b^Adjusted for clustering

Children stunted at the baseline survey had a significantly lower mean haemoglobin level (10.06 g/dl, 95% CI: 9.95–10.17 g/dl) than non-stunted children (10.72 g/dl, 95% CI: 10.66–10.79, *P* < 0.001) and were significantly more likely to be mildly or moderately anaemic (Table 4). Wasted and underweight children also had a significantly lower mean haemoglobin level at baseline than non-wasted and non-underweight children. Underweight children were significantly more likely to have mild (66.8%) or moderate anaemia (8.5%) than their non-underweight counterparts (mild: 55.5%, *P* < 0.001; moderate: 5.2%, *P* = 0.003). Children that were malnourished at the baseline survey were no more likely to be parasitaemic than non-malnourished children, with about 5% of children parasitaemic, irrespective of malnutrition status. At the end of season survey, stunted, wasted and underweight children had a significantly lower mean haemoglobin level than their well-nourished counterparts, and stunted and underweight children were significantly more likely to have mild or moderate anaemia. Parasitaemia prevalence at the end of the transmission season was similar between stunted and non-stunted, wasted and non-wasted and underweight or non-underweight children.

**Table 4:** Bi-variable analysis of prevalence of anaemia and parasitaemia by malnourished status from cross-sectional surveys at the start and end of the transmission season

Variable	Non-stunted children	Stunted children	*P*-value^a^	Non-wasted children	Wasted children	*P*-value^a^	Non-underweight children	Underweight children	*P*-value^a^
Beginning of malaria transmission season (June 2011)									
*N*	1715	796		2236	272		1892	624	
Mean haemoglobin (g/dl) (95% CI)	10.72 (10.66–10.79)	10.06 (9.95–10.17)	<0.001	10.54 (10.48–10.60)	10.34 (10.15–10.53)	0.04	10.63 (10.56–10.69)	10.18 (10.06–10.31)	<0.001
Mild anaemia (Hb < 11 g/dl) (*n*, %)	906 (52.8)	561 (70.5)	<0.001	1296 (58.0)	168 (61.8)	0.2	1051 (55.5)	417 (66.8)	<0.001
Moderate anaemia (Hb < 8 g/dl) (*n*, %)	68 (4.0)	83 (10.4)	<0.001	129 (5.8)	22 (8.1)	0.1	98 (5.2)	53 (8.5)	0.003
*P. falciparum* parasitaemia (*n*, %)	98 (5.7)	39 (4.9)	0.4	126 (5.6)	11 (4.0)	0.3	109 (5.8)	29 (4.6)	0.3
End of malaria transmission season (January 2012)^b^									
*N*	1427	725		1949	177		1721	437	
Mean haemoglobin (g/dl) (95% CI)	10.49 (10.41–10.58)	9.73 (9.60–9.85)	<0.001	10.26 (10.18–10.33)	9.96 (9.69–10.23)	0.03	10.37 (10.29–10.45)	9.72 (9.54–9.89)	<0.001
Mild anaemia (Hb < 11 g/dl) (*n*, %)	798 (55.9)	532 (73.4)	<0.001	1203 (61.7)	113 (63.8)	0.6	1024 (59.5)	307 (70.3)	<0.001
Moderate anaemia (Hb < 8 g/dl) (*n*, %)	91 (6.4)	116 (16.0)	<0.001	183 (9.4)	22 (12.4)	0.2	136 (7.9)	70 (16.0)	<0.001
Severe anaemia (Hb < 5 g/dl) (*n*, %)	2 (0.1)	2 (0.3)	0.5	4 (0.2)	0	0.5	2 (0.1)	2 (0.5)	0.1
*P. falciparum* parasitaemia (*n*, %)	179 (12.5)	108 (14.9)	0.1	259 (13.3)	22 (12.4)	0.7	224 (13.0)	62 (14.2)	0.5

^a^Two sided t-test or Chi-square test

^b^Children with *z*-scores of more than 4 or less than -4 not included and anthropometric data from children aged > 5 years at end of transmission survey were censored

**Fig. 1:**
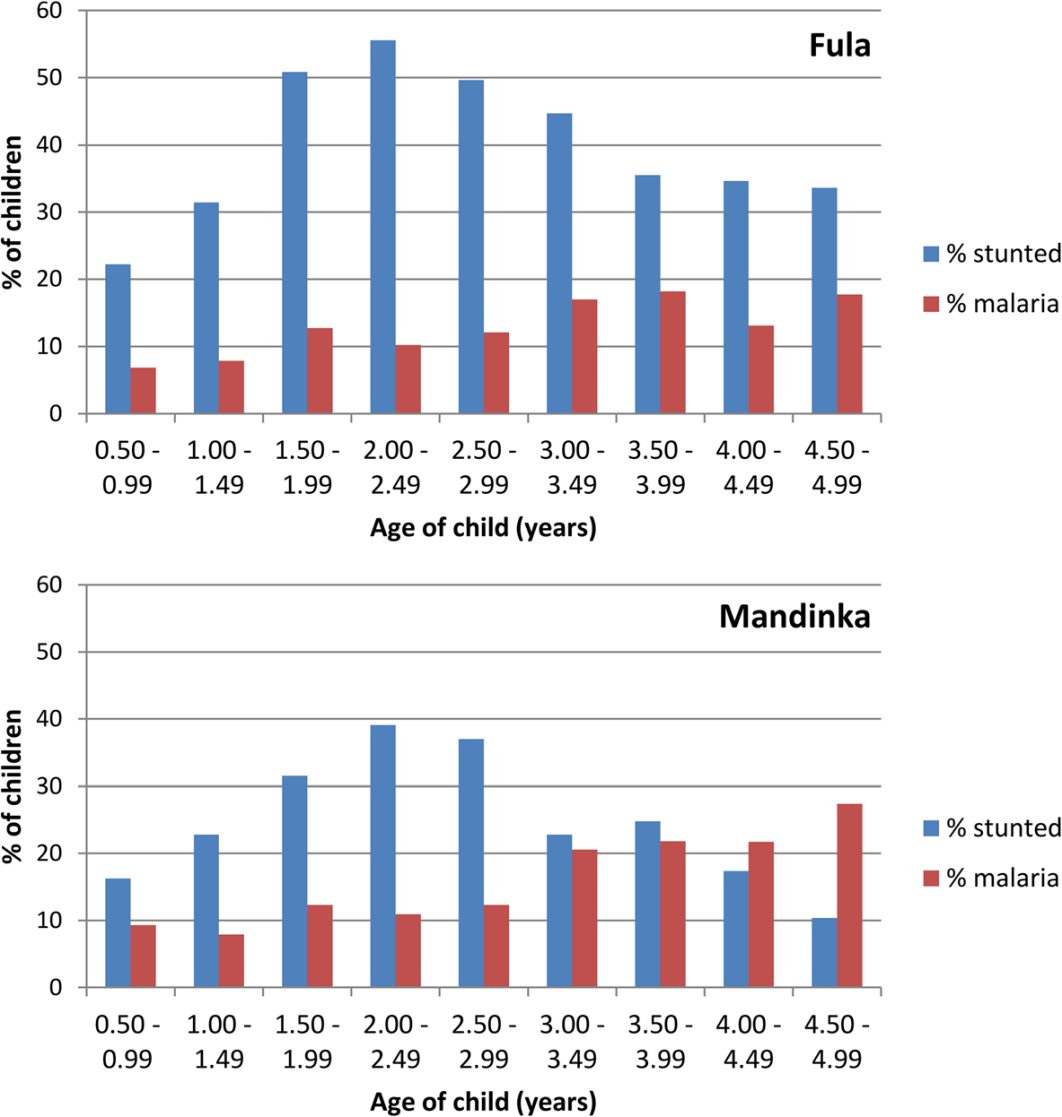
Prevalence of stunting at baseline survey and proportion of children suffering from one or more malaria episodes during transmission season by age group, and stratified by the most common ethnic groups: Fula and Mandinka

The risk of clinical malaria increased with age, while stunting increased steadily up to 2.0–2.5 years and then declined. Stunting was more common across all age groups in Fula than Mandinka children (Fig. 1). The decline in stunting from age 2.5 years onwards was less prominent in Fula children, with a high and steady stunting prevalence of over 30% in these age groups. In contrast, stunting decreased from 3 years of age in Mandinka children while their malaria risk increased.

During the transmission season, 13.7% of children (347/2527) in the cohort experienced at least one clinical malaria episode. Most of these episodes occurred in October (39%) and November (34%). In bi-variable analysis, stunting appeared mildly protective against clinical malaria (odds ratio, OR = 0.75, 95% CI: 0.57–0.98) (Table 5). However after adjusting for age, ethnicity and which bank of the river the village was located on, stunting was not associated with malaria (OR = 0.79, 95% CI: 0.60–1.05, *P* = 0.11). With every year of age gained, children had 1.4 times the odds of clinical malaria (95% CI: 1.27–1.55, *P* < 0.001) and those living on the north bank of the river had approximately half the odds of malaria compared to those living on the south bank (OR = 0.48, 95% CI: 0.29–0.79, *P* = 0.004). There was some indication that Mandinka were more likely to have malaria than Fula children, although the result was of borderline significance (OR comparing Mandinka to Fula children = 1.52, 95% CI: 0.98–2.36, *P* = 0.06). Sleeping under an LLIN and in a house that received IRS were not found to be associated with malaria risk.

**Table 5:** Multivariable analysis of risk of at least one malaria episode during the follow-up period by malnutrition and other factors measured at start of transmission season survey

Risk factor (*N* = 2527)	Malaria episode, *n* (%)	Odds ratio^b^ (95% CI)	*P*-value	Adjusted odds^c^ ratio (95% CI)	*P*-value
Stunting^a^					
No	259/1715 (15.1)	1		1	
Yes	88/796 (11.1)	0.75 (0.57–0.98)	0.04	0.79 (0.60–1.05)	0.11
Wasting^a^					
No	310/2,236 (13.9)	1			
Yes	33/272 (12.1)	0.85 (0.56–1.27)	0.42		
Underweight^a^					
No	268/1892 (14.2)	1			
Yes	77/624 (12.3)	0.93 (0.69–1.24)	0.61		
Mean age (years)	–	1.41 (1.28–1.56)	<0.001	1.40 (1.27–1.55)	<0.001
Gender					
Male	180/1348 (13.4)				
Female	167/1179 (14.2)	1.01 (0.79–1.29)	0.93		
Ethnicity					
Fula	139/1073 (13.0)	1		1	
Mandinka	195/1205 (16.2)	1.47 (0.95–2.28)	0.09	1.52 (0.98–2.36)	0.06
Other	13/245 (5.3)	0.44 (0.19–1.03)	0.06	0.52 (0.22–1.20)	0.13
Mean haemoglobin (g/dl)	–	1.03 (0.95–1.12)	0.44		
Mild anaemia					
No	157/1051 (14.9)	1			
Yes	190/1476 (12.9)	0.79 (0.62–1.02)	0.07		
Moderate anaemia					
No	319/2376 (13.4)	1			
Yes	28/151 (18.5)	1.40 (0.88–2.23)	0.16		
*P. falciparum* parasitaemia					
No	325/2388 (13.6)	1			
Yes	22/139 (15.8)	1.04 (0.62–1.73)	0.88		
Village location					
South bank	218/1224 (17.8)	1			
North bank	129/1303 (9.9)	0.47 (0.29–0.78)	0.003	0.48 (0.29–0.79)	0.004
House construction					
Modern	188/1454 (12.9)	1			
Traditional^b^	159/1073 (14.8)	1.08 (0.83–1.41)	0.55		
Slept under an LLIN					
No	2/16 (12.5)	1			
Yes	327/2366 (13.8)	1.07 (0.21–5.36)	0.94		
House received IRS-DDT					
No	176/1258 (14.0)				
Yes	171/1269 (13.5)	0.97 (0.57–1.64)	0.90		
Distance from health post or health facility (km)	–	1.03 (0.97–1.10)	0.34		

^a^Stunted *N* = 2511; Wasted *N* = 2508; Underweight *N* = 2516. These numbers differ from *N* = 2527 since children with *z*-scores of more than 4 or less than -4 were removed

^b^At least 50% of rooms with mud walls and thatched roof

^c^Adjusted for clustering

The mean weight gain between the two surveys among children who had at least one episode of clinical malaria (1.47 kg, 95% CI: 1.31–1.62) was similar to those that did not have malaria (1.54 kg, 95% CI: 1.40–1.68) (Table 6). Adjusting for confounders and clustering did not alter the result (regression β for mean weight at end of season survey -0.05, 95% CI: -0.44–0.33, *P* = 0.8). Similarly, there was no difference in height gain between children that had and those that did not have malaria (adjusted regression β for mean height at end of season survey -0.15, 95% CI: -0.68–0.38, *P* = 0.6). There was a reduction in mean haemoglobin concentration of 0.17 g/dl (95% CI: 0.11–0.22) among children who did not have malaria and 0.20 g/dl (95% CI: 0.07–0.33) in children who suffered from at least one malaria episode. After adjusting for confounders and clustering, children with malaria experienced significantly higher loss in haemoglobin concentration compared to children who did not have malaria over the transmission season [adjusted regression β (95% CI) for mean haemoglobin at end of season survey -0.17, 95% CI: -0.03– -0.31, *P* = 0.02].

**Table 6:** Multivariable analysis of changes in weight, height and haemoglobin concentration among children who experienced at least one malaria episode and those that were malaria-free during the transmission season

	Child without a malaria episode	Child with at least one malaria episode
Mean weight at first survey (kg) (95% CI)	11.46 (11.34–11.58)	12.50 (12.21–12.80)
Mean weight at second survey (kg) (95% CI)	13.00 (12.82–13.18)	13.96 (13.65–14.28)
Mean difference in weight (kg) (95% CI)	1.54 (1.40–1.68)	1.47 (1.31–1.62)
Adjusted regression β (95% CI) for mean weight at second survey^a^	Reference	-0.05 (-0.44–0.33)
*P*-value	–	0.8
Mean height at first survey (cm) (95% CI)	86.8 (86.4–87.3)	91.5 (90.4–92.7)
Mean height at second survey (cm) (95% CI)	91.3 (90.9–91.8)	95.6 (94.4–96.8)
Mean difference in height (cm) (95% CI)	4.7 (4.6–4.9)	4.4 (3.7–5.0)
Adjusted regression β (95% CI) for mean height at second survey^a^	Reference	-0.15 (-0.68–0.38)
*P*-value	–	0.6
Mean haemoglobin at first survey (g/dl) (95% CI)	10.51 (10.45–10.58)	10.53 (10.36–10.70)
Mean haemoglobin at second survey (g/dl) (95% CI)	10.37 (10.29–10.44)	10.35 (10.17–10.54)
Mean difference in haemoglobin (g/dl) (95% CI)	-0.17 (-0.11– -0.22)	-0.20 (-0.07– -0.33)
Adjusted regression β (95% CI) for mean haemoglobin at second survey^a^	Reference	-0.17 (-0.03– -0.31)
*P*-value		0.02

^a^Adjusted for weight, height or haemoglobin at first survey, gender, age, sleeping under an LLIN, IRS, ethnicity, traditional house, river bank and clustering

## Discussion

Malnutrition, in particular chronic malnutrition (stunting), was common in this cohort of children, with a third of them stunted at the end of the malaria transmission season. This corroborates the results of the 2010 Multiple Indicator Cluster Survey (MICS) which shows a 35.6% stunting prevalence in the URR [34]. Similar surveys show that the prevalence of stunting in the URR increased from 29.4% in 2000 to 34.6% in 2006, indicating a persisting problem [35, 36]. In our study, stunting was very common in Fula children and in those living in a traditional house. Although stunted and underweight children tended to be anaemic, they did not have a higher risk of *P. falciparum* infection and stunting did not appear to be associated with clinical malaria in this cohort. On average, children tended to increase in weight and height over the course of the transmission season and malaria did not have any influence over these nutritional variables.

Our analysis identified several risk factors for malnutrition in the URR including ethnicity and poor housing, the latter finding suggesting that, as would be expected, chronic malnutrition is associated with poverty [37, 38]. Feeding practices are known to be poor in The Gambia, with relatively few children being fed according to international guidelines, including breastfeeding until two years of age and the introduction of solid and semi-solid foods at six months [38]. In particular, the Fula have specific feeding practices which may be detrimental to maternal and child nutrition. Historically nomadic pastoralists, Fula have become increasingly sedentary in The Gambia, including those families in this study. Milk production and consumption is very low in The Gambia. The Fula keep cattle largely as a symbol of wealth and there is little nutritional benefit gained from their husbandry. Maternal malnutrition can also contribute to childhood malnutrition, with data from The Gambia showing that women with a low body mass index are more likely to have stunted children [38]. Fula in the URR have been shown to have food taboos which can contribute to reduced protein and calorific intake [39]. For example, pregnant Fula women typically do not consume eggs, bread, bananas, catfish or groundnut. Fula women are also likely to feed only after the men and children have eaten due to gender dynamics within the household [39].

Similar to other surveys in The Gambia [4, 9, 38, 40], stunting was more common in children aged around two years than in younger or older children. After birth, infants tend to gain weight quickly due to breast-feeding and are largely protected from infections due to protective maternal antibodies. As children are weaned and passive immunity fades, malnutrition and infections become more common. From about two years, the process of stunting slows down and growth improves as children accept the prevalent diet and acquired immunity increases [41].

Stunting and underweight were strongly associated with anaemia at both the beginning and end of the malaria transmission season. This is an important finding since anaemia is a major cause of mortality in children aged under five years of age [42]. The aetiology of childhood anaemia in this setting is likely to be multifactorial including iron deficiency and infectious diseases such as malaria and some helminth infections [43].

There was no association between malnutrition and *P. falciparum* infection at either cross-sectional survey, nor was there an association between malnutrition (stunting, wasting or underweight) at the baseline survey and malaria incidence during the transmission season. This finding is supported by a systematic review of observational studies which concluded that there was no association between anthropometric parameters and malaria incidence [21]. Although the review found no overall association, there were some discrepant results between studies. Reasons given for this by Ferreira et al. [21] were possibly differences in diagnostic techniques used for malaria and nutritional status, lack of adjustment for relevant confounders and differences in duration of follow up. Studies that showed a protective effect of malnutrition on malaria had at least one year of follow up while those showing malnutrition as a malaria risk factor typically had six months or less of follow up. Despite the systematic review not showing an association between malnutrition and malaria in community studies, there was evidence that malnutrition increases the risk of severe malaria and malaria case fatality rate in hospitalised children [21], including one study from The Gambia [44].

There was indication of a higher clinical malaria incidence in Mandinka compared to Fula children, although the result was of borderline significance. Previous studies have shown conflicting results with Greenwood et al. [45] reporting that malaria and splenomegaly were less common in Mandinkas than Wolofs or Fula in The Gambia and other studies showing lower parasite rate in Fula compared to sympatric ethnic groups [46–48], perhaps due to differences in immune function and response among Fula [49]. We found that children living on the south bank were more likely to have malaria compared to children living on the north bank of the River Gambia. This was unexpected given that the south bank is more affluent than the north bank. However, since the study used passive case detection, the result may reflect differences in treatment seeking if caregivers on the south bank were more likely to attend a health facility, perhaps facilitated by better roads and public transport. We did not find an association between anaemia at the baseline survey and malaria in the multivariable logistic regression model suggesting that anaemia did not predispose children to malaria. There was no difference in malaria incidence between children who slept under a LLIN and those that did not, probably as a result of small numbers of children not using an LLIN in this study. We also did not find an association between IRS and malaria incidence. This corresponds to the results of the original trial which showed no difference in malaria between the IRS-LLIN and LLIN study arms [31].

Malaria did not have any influence on weight and height gain during the malaria transmission season. Two recent systematic reviews concluded that there was no association between malaria and malnutrition [21, 25]. However, studies show conflicting results for this association [9, 22, 50] and several intervention studies show beneficial effects of malaria control on nutritional status, including weight gain [26, 27, 51, 52]. Therefore, it is possible that the seven month period between the two surveys in our study was too short to determine any difference in weight or height gain. Since most malaria episodes occurred in a very short period (October and November 2011) and the survey was carried out only two months later (January 2012), the period might not have been sufficient to detect any difference, which may appear only after several months. Secondly, easy access to diagnosis and treatment of malaria episodes during the study may have prevented the prolonged carriage of *P. falciparum* and its associated growth suppressing immune response which could have led to a lack of differences between the two groups of children. Lastly, changes in feeding or other health behaviours directed towards children who were sick with malaria in order to aid their recovery may also have impacted on their weight or height gain between the two surveys. Children who had malaria during the transmission season showed significantly greater reductions in haemoglobin concentration than those who did not suffer from malaria. This was unsurprising since blood stage infection with malaria parasites induces anaemia. The reduction in haemoglobin concentration over the transmission season also seen among children not suffering from malaria suggests possible nutritional deficiency or inflammation/infection, including perhaps asymptomatic malaria infection [53].

Observational study designs such as that employed are limited in the extent to which we can determine causal relationships due to possible biases and residual confounding. The study did not measure factors such as diet, micronutrient status and other infections such as diarrhoea which can confound the relationship between malaria and stunting [12, 54]. There also may have been residual confounding due to socioeconomic status since this was not measured in the study, only proxies such as housing construction. Although the study used passive case detection, we consider that few malaria cases would have been missed. This is because cases were passively detected at both primary health posts staffed by village health workers and secondary health care facilities and most of the study population lived within close proximity to one of these two types of facility (median distance 3 km, interquartile range 0 and 6 km). Study staff worked alongside government-appointed primary and secondary health care staff providing training, support and monitoring case records. There was also no cost to access care for the study participants since malaria diagnosis and treatment was provided free of charge and any travel costs incurred were reimbursed by study staff.

## Conclusions

While stunting was not a risk factor for malaria in this study, the high levels of stunting and anaemia identified, particularly among the Fula, require urgent attention. Poverty leading to insufficient food or lack of variety of food, poor status of women, absence of a support network, and alternative cultural beliefs about the origin and treatment of malnutrition have been identified as contributing to poor child nutrition practices in The Gambia [37]. Malnutrition tends to decrease as countries develop [55]. However, studies aiming to determine the specific package of interventions required to prevent malnutrition have frequently failed. For example, data from Keneba in the Lower River Division of The Gambia suggest that despite numerous public health interventions and increased food security, reductions in malnutrition have been stubbornly slow [40, 56]. Even in Keneba, which is more affluent and has much better access to health services than the URR, stunting was 30% in the period from 2000 to 2012 [40]. This suggests that there is a high threshold of development which needs to be achieved to impact malnutrition, including perhaps household-level rather than village-level water pipe provision [40]. Efforts will need to be strengthened in order to reach Sustainable Development Goal 2 “to end hunger, achieve food security and improved nutrition, and promote sustainable agriculture”, and in particular, indicators on stunting and wasting in children under five years of age [57].

## Abbreviations

CI: Confidence interval
Hb: Haemoglobin
IRS: Indoor residual spraying
LLIN: Long-lasting insecticidal net
MICS: Multiple Indicator Cluster Survey
OR: Odds ratio
SSA: Sub-Saharan Africa
URR: Upper River Region
WHO: World Health Organization
